# *Helicobacter pylori* Infection and Risk of Lung Cancer: A Meta-Analysis

**DOI:** 10.1155/2013/131869

**Published:** 2013-02-28

**Authors:** Pulikonda Mounika

**Affiliations:** Vasavi College of Pharmacy, Tadepalligudem, Andhra Pradesh 534101, India

## Abstract

*Background*. Recent evidence showed that *Helicobacter pylori* seropositivity is a risk factor for gastric and several other cancers. However, evidence on *H. pylori* infection and risk of lung cancer has been controversial, with a limited number of underpowered studies. We therefore examined the association between *H. pylori* infection and risk of lung cancer. *Methods*. A comprehensive literature search was performed using PubMed, EMBASE (until October 2012) for studies investigating an association between *Helicobacter pylori* (*H. pylori*) infection and risk of lung cancer. Pooled odds ratio (OR) was calculated using random-effects model. Subgroup and sensitivity analysis were also done. *Results*. A total of seven studies (6 case-control and 1 cohort study) were included for the analysis. There was a significant heterogeneity among the studies, but no publication bias was observed. We found that *H. pylori* infection was associated with significantly increased risk of lung cancer (pooled OR, 2.29 (95% CI, 1.34–3.91) *P* = 0.01). *Conclusions*. Our meta-analysis suggests a significant increased risk of lung cancer in patients with *H. pylori* infection. Further research is needed to confirm these findings and to identify the underlying biological mechanisms.

## 1. Introduction

Lung cancer is the second most common cancer in both men and women. Most recent estimates of American Cancer Society reflect 160,340 deaths due to lung cancer (87,750 in men and 72,590 in women), accounting for about 28% of all cancer deaths in United States [1].

*Helicobacter pylori *(*H. pylori*) is one of the most common bacterial infections of humans affecting approximately 50% of the world's population [2]. This Gram-negative bacterium infects the human gastric mucosa and causes long-term colonization and inflammation. In a subpopulation of infected individuals, long-term inflammation results in peptic ulcer disease and gastric malignancy [3]. Recent evidence showed that *H. pylori* seropositivity is also a risk factor for gastric [4], colorectal [5], pancreas [6], and hepatobiliary cancers [7, 8]. An increased seroprevalence was also found in various respiratory diseases like chronic bronchitis [9], asthma [10], and pulmonary tuberculosis [11].

However, evidence on *H. pylori* infection and risk of lung cancer has been controversial, with a limited number of underpowered studies that report result of increased risk [12–14], decreased risk [15], or no association [3, 16, 17] between the *H. pylori* infection and risk of lung cancer. This issue was discussed in previously conducted meta-analysis that analyzed the risk of lung cancer due to *H. pylori* infection by including 4 underpowered case-control studies published between 2000 and 2007 [18]. They concluded that there is an increased risk of lung cancer by 3.26 times due to *H. pylori* infection (confidence interval was wider to give consolidate conclusion). However, three more studies (1 case-control and 2 large prospective studies) evaluating the association between *H. pylori* infection and lung cancer were published after 2007 [3, 12, 15]. In this updated meta-analysis, we examined the association between *H. pylori* infection and risk of lung cancer. 

## 2. Materials and Methods

### 2.1. Literature Search

A comprehensive literature search was performed using PubMed, EMBASE, covering all published papers until October 2012 with a combination of the following keywords: lung cancer, *Helicobacter pylori*, *H. pylori*, *Campylobacter pylori*, and peptic ulcer disease with limits; humans and English. 

The author evaluated potentially associated publications by checking their titles and abstracts and then procured the most relevant publications for a closer examination. Bibliographies section of retrieved articles was also reviewed for additional pertinent studies that possibly were missed in the initial search. 

### 2.2. Inclusion and Exclusion Criteria

Studies were included if (1) it is an observational study; (2) its objective of interest is assessing the association of *H. pylori* infection with lung cancer risk; (3) it mentions the method of lung cancer diagnoses and sources of cases and controls; (4) it mentions the sample size and effect estimates.

Articles were excluded if they were reviews, letters to the editor without original data, editorials, and case reports. The paper were also excluded if no effect estimates were reported or not enough raw data for an odds ratio to be calculated. We reviewed all papers in accordance with the criteria defined above for further analysis.

### 2.3. Data Extraction

Full text of probable studies was retrieved and reviewed to assess the appropriateness for inclusion in the present meta-analysis. The following data were extracted from each study: (a) first author's last name, year of publication, and country of the population studied; (b) study design; (c) number of lung cancer cases; (d) number of *H. pylori* infected patients; (e) incidence rates or effect estimate with 95% confidence interval; (f) source of study population; (g) lung cancer and *H. pylori* infection assessment.

### 2.4. Quality Assessment

The quality of each included study was assessed by using the Newcastle-Ottawa Scale (NOS) [19]. The NOS assigns a maximum of four points for selection: two points for comparability and three points for exposure/outcome. Therefore, studies of the highest and medium quality reflect 9 and 7 or 8 points, respectively. Any discrepancies were addressed by a joint revaluation of the original article with a third author.

### 2.5. Data Synthesis and Analysis

The primary measure was odds ratio (OR) of lung cancer, calculated using the random-effects model (DerSimonian and Laird method), which accounts for heterogeneity among studies. To assess heterogeneity among the studies, we used the Cochran *Q* and *I* ^2^ statistics; for the *Q* statistic, a *P* value <0.10 was considered statistically significant for heterogeneity; for *I* ^2^, a value >50% is considered a measure of severe heterogeneity [20]. 

Prespecified subgroup analysis was performed to assess the source of heterogeneity, according to (a) study design, (b) histology of lung cancer, (c) quality scale (NOS), and (iv) studies before and after the Zhuo et al. [18] analysis. To assess the robustness of the association, we also performed sensitivity analysis by excluding the outliers. The publication bias was assessed using funnel plot and Begg and Mazumdar adjusted rank correlation test [21, 22]. All statistical tests were two-sided, and *P* < 0.05 was considered statistically significant, except where otherwise specified. Data were analysed using Comprehensive Meta-Analysis software. The present work was performed as per the guidelines proposed by the Meta-analysis of Observational Studies in Epidemiology group [23] and Preferred Reporting Items for Systematic Reviews and Meta-Analyses (PRISMA) (Checklist S1 in the supplementary material available online at http://dx.doi.org/10.1155/2013/131869). 

## 3. Results

**Figure 1:**
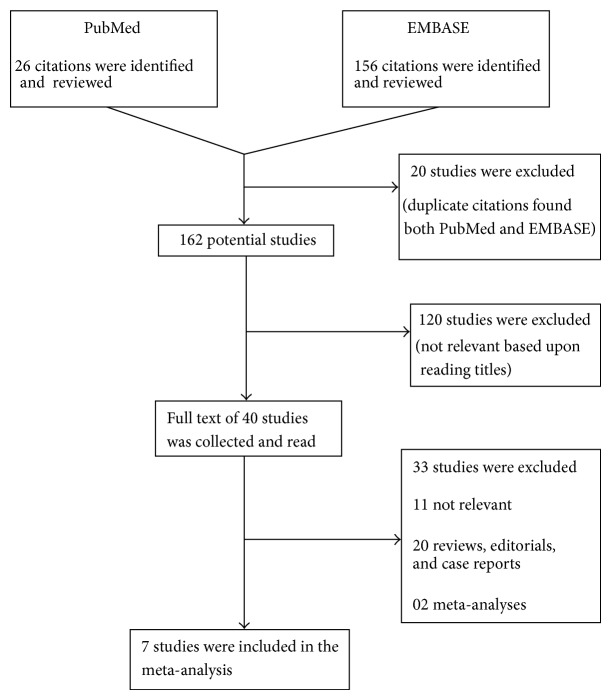
Flowchart representing selection process.

Search results were shown in Figure 1.

### 3.1. Study Characteristics

 Seven relevant studies were identified including 5 case-control and 2 prospective (1 nested case-control [3] and 1 cohort [15]) studies involving a total of 16,244 lung cancer cases and 1,707 *H. pylori* infection patients. These studies were published between 2000 and 2007. The follow-up period for the cohort study was about 10 years. All studies assessed the *H. pylori* infection by Enzyme-Linked-Immuno sorbent Assay. Five studies histologically confirmed lung cancer [13–17], whereas one study assessed by checking the health register [3]. Only one study reported the subtype of lung cancer based on histology (lung adenocarcinoma and squamous cell carcinoma) [3]. Characteristics of the included studies are presented in Table 1.

### 3.2. Quality Assessment Results

When the quality of the included studies was assessed, 1 high [3], 4 medium [14–17], and 2 low, quality studies were found [12, 13] (Table 1). Cohort study had a NOS score of 7. With regard to case-control studies, only one study had NOS score of 9. Together case-control studies have an average NOS score of 8.4.

### 3.3. Main Analysis

**Figure 2:**
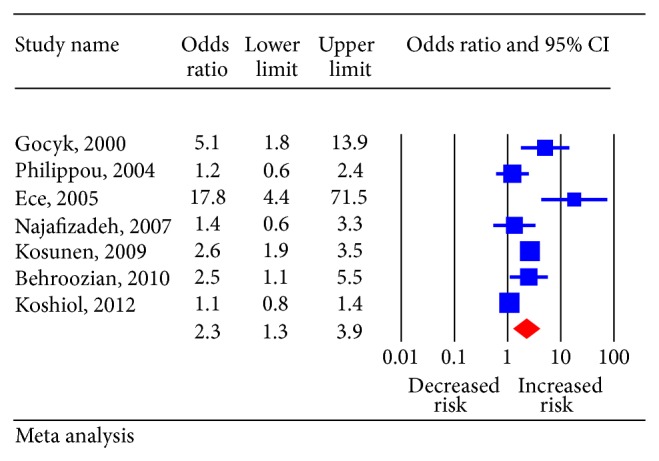
Pooled estimate of odds ratio (OR) and 95% confidence intervals (CIs) of risk of lung cancer in *Helicobacter pylori* infected patients. Squares indicate OR in each study. The square size is proportional to the weight of the corresponding study in the meta-analysis; the length of horizontal lines represents the 95% CI. The diamond indicates the pooled OR and 95% CI (random-effects model).

**Figure 3:**
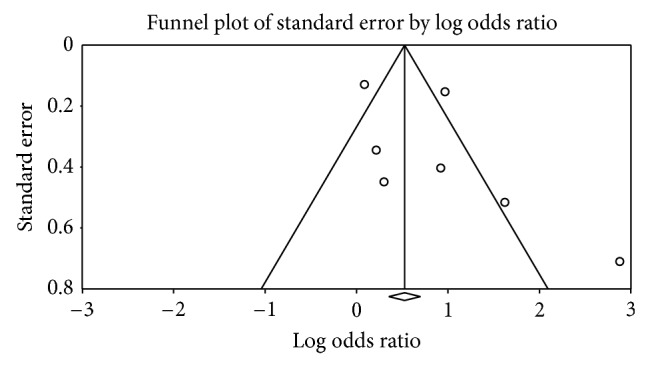
Funnel plot (publication bias assessment plot) of the odds ratio of lung cancer, by the standard error, for all studies. Circles: studies in the meta-analysis. Odds ratios are displayed on logarithmic scale.

Because a significant heterogeneity was found (*P*_heterogeneity_ < 0.01, *I* ^2^ = 83.9%), random-effects model was chosen over a fixed-effect model. We found that *H. pylori* infection was associated with significantly increased risk of lung cancer (pooled OR, 2.29 (95% CI, 1.34–3.91) *P* = 0.01). The ORs of lung cancer for each study and all studies combined are shown in Figure 2. Visual examination of the funnel plot revealed minimal asymmetry (Figure 3), further confirmed by Begg's test (*P* = 0.22) indicating little or no publication bias in our analysis.

### 3.4. Sensitivity Analysis

Sensitivity analysis showed a significant variation in pooled OR from 2.29 to 1.88 (95% CI, 1.14–3.18) after removing the study by Ece et al. [13], (this study had significantly wider confidence interval compared to all other studies). However, the estimated effect size did not deviate much by excluding any of the other studies one at a time (OR between 2.06–2.68) (Supplemental Figure 1).

### 3.5. Subgroup Analysis

Results of subgroup analysis were shown in Table 2. We found a significant positive association between *H. pylori* infection and risk of lung cancer in both case-control studies (OR 2.28 (95% CI, 1.19–4.39)) and one cohort study (OR 2.63 (95% CI, 1.95–3.55)). There is no significance in association according to study design. Zhuo et al. [18] reported an OR 3.11 (95% CI, 1.07–9.04) by pooling four studies.Three studies were published after the Zhuo et al. meta-analysis. Pooled OR of these three studies is 1.87 (95% CI, 0.93–3.37). There is a significant difference between the studies published before and after the Zhuo et al. [18] meta-analysis. Only one study reported subtype of lung cancer based on histology that showed risk of 1.1 (95% CI, 0.75–1.6) for lung adenocarcinoma and 1.1 (95% CI, 0.77–1.7) for lung squamous cell carcinoma. There was one study with high quality which showed OR 1.09 (95% CI, 0.85–1.41) [3]. Four medium quality studies showed OR 2.12 (95% CI, 1.24–3.61) [14–17].

## 4. Discussion

The present updated pooled analysis of 7 studies currently available showed that patients with *H. pylori* infection were associated with an estimated 2.3 times increased risk of developing lung cancer as compared with those without *H. pylori* infection. Present analysis included three large sample studies when compared to the previous meta-analysis [18]. Present analysis reported a OR 2.34 (95% CI 1.34–4.07) which is based on the inclusion of 16,244 lung cancer cases and 1,707 *H. pylori* infection patients. Thus power of the result is increased.

Reason for the increased risk of lung cancer in *H. pylori* infected patients can be explained in several ways. (i) *H. pylori* is a Gram-negative bacteria with lipopolysaccharide as the major component of the cell wall. Lipopolysaccharide stimulates the production of proinflammatory cytokines including interleukins and tumor necrosis factor-alpha [24]. This leads to chronic inflammation and immune stimulation, which may contribute to carcinogenesis [9, 25, 26]. (ii) The lungs arise embryological from the same endoderm cells that form the lining of the gastrointestinal tract and possess similar neuroendocrine and paracrine cells releasing various hormonal peptides and their receptors including gastrin-releasing peptide and gastrin [27]. It is a well-known fact that *H. pylori* infection in the stomach markedly enhances and prolongs the release of gastrin [14]. Gocyk et al. showed that gastric *H. pylori* infection in lung cancer patients is accompanied by a significant increase in gastrin plasma and bronchial lavage levels as well as by increased mRNA expression for gastrin and its receptors, as well as for Cyclo oxygenase-1 (COX-1) and COX-2 in the tumor tissue [14]. Gastrin could contribute to lung cancer by inducing higher mucosal cell proliferation of bronchial epithelium to atrophy and induction of COX-2.

We found no significant difference between the cohort and case-control studies, though there was only one cohort study. Three studies published after Zhuo et al. showed a nonsignificant increased risk (OR, 1.87 (0.93–3.37)) of lung cancer in *H. pylori* infected patients [3, 12, 15]. These three studies had a larger sample size than the reported earlier studies; this leaves us to a dilemma that there may be no probable association between *H. pylori* infection and lung cancer. Koshiol et al. in a large case-control study showed no association between *H. pylori* infection and risk of lung adenocarcinoma (OR, 1.1 (0.75–1.6)) and also lung squamous cell carcinoma (OR, 1.1 (0.77–1.7)) [3].

The strength of the present meta-analysis lies in inclusion of 7 observational studies reporting data on 16,244 lung cancer cases and 1,707 *H. pylori* patients. Our meta-analysis has several limitations. First, most of the included studies are underpowered with less number of study subjects. Secondly only one study has reported confounder adjustment like smoking, which is one of the potential risk factors of lung cancer. Other limitations include not searching for unpublished studies for original data. Finally, our analysis was restricted to articles in the English language. 

In summary, our results suggest a significant increased risk of lung cancer in patients with *H. pylori* infection. However, the result should be cautiously interpreted due to the inclusion of underpowered studies. Further large prospective studies are needed to address the association of *H. pylori* infection and lung cancer and its subtypes according to histology. 

## Supplementary Material

PRISMA checklist contains 27 checklist items pertain to the content of a systematic review which helps in improving the reporting of systematic reviews and meta-analyses. This supplementary material consists of checklist filled with page numbers of the required items to assess the reporting of meta-analysis.

## Figures and Tables

**Table 1:** Characteristics of the included studies on *Helicobacter pylori* infection and risk of lung cancer.

First author, year	Country	Diagnosis		Study size	Number		QR
		*H. pylori* infection	Lung cancer		Cases (*H. pylori* +)	Controls (*H. pylori* +)	
Gocyk, 2000 [14]^#^	Poland	A	A	150	50 (45)	100 (64)	7
Philippou, 2004 [17]^#^	Greece	A	A	140	72 (44)	68 (38)	7
Ece, 2005 [13]^#^	Turkey	A	A	71	43 (40)	28 (12)	6
Najafizadeh, 2007 [16]^#^	Iran	a	A	80	34 (21)	35 (18)	8
Kosunen, 2009 [15]∗	Finland	a	NR	26,705	NA	NA	7
Behroozian, 2010 [12]^#^	Iran	a	A	132	66 (48)	66 (34)	5
Koshiol, 2012 [3]^$^	Finland	a	B	1,389	696 (550)	693 (544)	9

^#^Case-control study; ∗prospective study; ^$^nested case-control study.

NR: not reported; NA: not applicable; QR: quality rating according to New castle-Ottawa Scale;

*H. pylori *+: number of patients with seropositivity of *H. pylori.*

a: Enzyme-Linked Immunosorbent Assay.

A: histologically conformed; B: registry based.

**Table 2:** Overall effect estimates for *Helicobacter pylori* infection and lung cancer according to study characteristics.

Study	No. of studies	Random-effects model:	Heterogeneity between studies	
		Overall OR (95% CI)	*P* value^a^	*I* ^2^ value
All	7	2.29 (1.34–3.91)	<0.01	83.9%
Sensitivity analysis				
All except study by Ece et al. [13]^b^	6	1.88 (1.14–3.18)	<0.01	80.9%
Study design				
Cohort	1	2.63 (1.95–3.55)	NA	NA
Case-control	6	2.28 (1.19–4.39)	<0.01	79.9%
Time of Zhuo et al. [18]^c^				
Published in time frame covered in Zhuo et al. [18]	4	3.11 (1.07–9.04)	<0.01	80.9%
Published after Zhuo et al. [18]	3	1.87 (0.93–3.37)	<0.01	90.1%
Histology of lung cancer				
Lung adenocarcinoma	1	1.1 (0.75–1.6)	NA	NA
Lung squamous cell carcinoma	1	1.1 (0.77–1.7)	NA	NA
Quality of included studies^d^				
High quality	1	1.09 (0.85–1.41)	NA	NA
Medium quality	4	2.12 (1.24–3.61)	0.05	61.1%
Low quality	2	6.12 (0.90–41.38)	0.01	82.6%

OR: Odds ratio; CI: confidence interval; NA: not available.

^a^ *P* value obtained by Cochrane *Q* test.

^b^Ece et al. [13] is the one of the included studies which has wider confidence interval.

^c^Zhuo et al. [18] is the recent meta-analysis done on this subject.

^d^Quality of-included studies was assessed using Newcastle-Ottawa Scale.

## References

[1] American Cancer Society Cancer facts and figures 2012. http://www.cancer.org/acs/groups/content/@epidemiologysurveilance/documents/document/acspc-031941.pdf.

[2] Sachs G., Scott D. R. (2012). *Helicobacter pylori*: eradication or preservation. *F1000 Medicine Reports*.

[3] Koshiol J., Flores R., Lam T. K. (2012). *Helicobacter pylori* seropositivity and risk of lung cancer. *PLoS ONE*.

[4] Cavaleiro-Pinto M., Peleteiro B., Lunet N., Barros H. (2011). *Helicobacter pylori* infection and gastric cardia cancer: systematic review and meta-analysis. *Cancer Causes and Control*.

[5] Zumkeller N., Brenner H., Zwahlen M., Rothenbacher D. (2006). *Helicobacter pylori* infection and colorectal cancer risk: a meta-analysis. *Helicobacter*.

[6] Trikudanathan G., Philip A., Dasanu C. A., Baker W. L. (2011). Association between *Helicobacter pylori* infection and pancreatic cancer. A cumulative meta-analysis. *Journal of the Pancreas*.

[7] Pandey M., Mishra R. R., Dixit R., Jaiswal R., Shukla M., Nath G. (2010). Helicobacter bilis in human gallbladder cancer: results of a case-control study and a meta-analysis. *Asian Pacific Journal of Cancer Prevention*.

[8] Selgrad M., Bornschein J., Rokkas T. *Helicobacter pylori*: gastric cancer and extragastric intestinal malignancies. *Helicobacter*.

[9] Kanbay M., Kanbay A., Boyacioglu S. (2007). *Helicobacter pylori* infection as a possible risk factor for respiratory system disease: a review of the literature. *Respiratory Medicine*.

[10] Wang Y., Bi Y., Zhang L., Wang C. (2012). Is helicobacter pylori infection associated with asthma risk? A meta-analysis based on 770 cases and 785 controls. *International Journal of Medical Sciences*.

[11] Perry S., De Jong B. C., Solnick J. V. (2010). Infection with *Helicobacter pylori* is associated with protection against tuberculosis. *PLoS ONE*.

[12] Behroozian R., Moradkhan E. (2010). The assessment of probable relationship between lung cancer and *Helicobacter pylori* infection. *Tropical Gastroenterology*.

[13] Ece F., Hatabay N. F., Erdal N., Gedik C., Guney C., Aksoy F. (2005). Does *Helicobacter pylori* infection play a role in lung cancer?. *Respiratory Medicine*.

[14] Gocyk W., Nikliński T., Olechnowicz H. (2000). *Helicobacter pylori*, gastrin and cyclooxygenase-2 in lung cancer. *Medical Science Monitor*.

[15] Kosunen T. U., Pukkala E., Sarna S., Seppala K., Aromaa A. Does eradication of *H. pylori* infectyion delay the development lung cancer?.

[16] Najafizadeh K., Falah Tafti S., Shiehmorteza M., Saloor M., Jamali M. (2007). *H pylori* seroprevalence in patients with lung cancer. *World Journal of Gastroenterology*.

[17] Philippou N., Koursarakos P., Anastasakou E. (2004). *Helicobacter pylori* seroprevalence in patients with lung cancer. *World Journal of Gastroenterology*.

[18] Zhuo W., Zhu B., Xiang Z., Zhuo X., Cai L., Chen Z. (2009). Assessment of the relationship between *Helicobacter pylori* and lung cancer: a meta-analysis. *Archives of Medical Research*.

[19] Ottawa Hospital Research Institute The Newcastle-Ottawa Scale (NOS) for assessing the quality of nonrandomised studies in meta-analyses. http://www.ohri.ca/programs/clinical_epidemiology/oxford.asp.

[20] Higgins J. P. T., Thompson S. G., Deeks J. J., Altman D. G. (2003). Measuring inconsistency in meta-analyses. *British Medical Journal*.

[21] Begg C. B., Mazumdar M. (1994). Operating characteristics of a rank correlation test for publication bias. *Biometrics*.

[22] Egger M., Smith G. D., Schneider M., Minder C. (1997). Bias in meta-analysis detected by a simple, graphical test. *British Medical Journal*.

[23] Stroup D. F., Berlin J. A., Morton S. C. (2000). Meta-analysis of observational studies in epidemiology: a proposal for reporting. *Journal of the American Medical Association*.

[24] Thalmaier U., Lehn N., Pfeffer K., Stolte M., Vieth M., Schneider-Brachert W. (2002). Role of tumor necrosis factor alpha in *Helicobacter pylori* gastritis in tumor necrosis factor receptor 1-deficient mice. *Infection and Immunity*.

[25] Arnold I. C., Dehzad N., Reuter S. (2011). *Helicobacter pylori* infection prevents allergic asthma in mouse models through the induction of regulatory T cells. *Journal of Clinical Investigation*.

[26] Yokota S., Okabayashi T., Rehli M., Fujii N., Amano K. (2010). *Helicobacter pylori* lipopolysaccharides upregulate toll-like receptor 4 expression and proliferation of gastric epithelial cells via the MEK1/2-ERK1/2 mitogen-activated protein kinase pathway. *Infection and Immunity*.

[27] Frankel A., Tsao M., Viallet J. (1994). Receptor subtype expression and responsiveness to bombesin in cultured human bronchial epithelial cells. *Cancer Research*.

