# Supplementary figures and images for: Helicobacter pylori Infection and Risk of Lung Cancer: A Meta-Analysis
Source: Lung Cancer Int. 2013 Feb 28;2013:131869. doi: 10.1155/2013/131869 (PMC4437409; doi:10.1155/2013/131869)

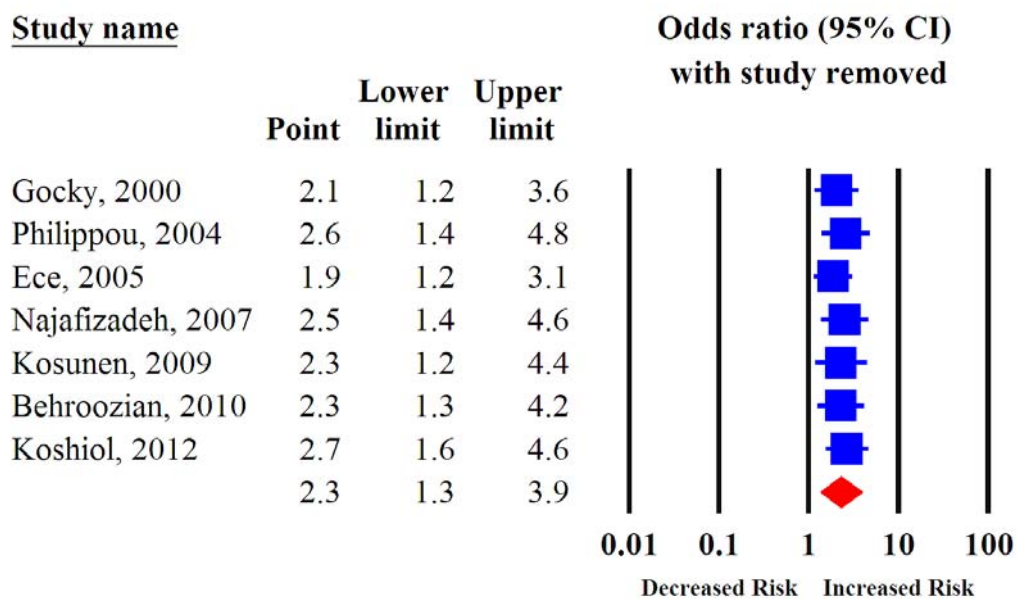

Meta Analysis

Supplement: Supplementary file 1 — PRISMA checklist contains 27 checklist items pertain to the content of a systematic review which helps in improving the reporting of systematic reviews and meta-analyses. This supplementary material consists of checklist filled with page numbers of the required items to assess the reporting of meta-analysis. [file 131869.f1.pdf]
